# Study on design optimization of GFRP tubular column composite structure based on machine learning method

**DOI:** 10.1371/journal.pone.0301865

**Published:** 2024-04-26

**Authors:** Peiyao Shu, Chengqi Xue, Gengpei Zhang, Tianyi Deng

**Affiliations:** Electronics and Information School, Yangtze University, Jingzhou City, Hubei Province, China; University of Duhok, IRAQ

## Abstract

Circular reinforced concrete wound glass fiber reinforced polymer (GFRP) columns and reinforced concrete filled GFRP columns are extensively utilized in civil engineering practice. Various factors influence the performance of these two types of GFRP columns, thereby impacting the whole project. Therefore, it is highly significant to establish the prediction models for ultimate displacement and ultimate bearing capacity to optimize the design of the two types of GFRP columns. In this study, based on the experiments conducted under different conditions on the two kinds of GFRP columns, automatic machine learning along with four other commonly used machine learning methods were employed for modeling to analyze how the column parameters (cross section shape, concrete strength, height of GFRP column, wound GFRP wall thickness, inner diameter of wound GFRP column) affect their performance. The differences in performance among these five machine learning methods were analyzed after modeling. Subsequently, we obtained the variation patterns in ultimate displacement and ultimate bearing capacity of the columns influenced by each parameter by testing the data using the optimal model. Based on these findings, the optimal design schemes for the two types of GFRP columns are proposed. The contribution of this paper is three-fold. First, AutoML sheds light on the automatic prediction of ultimate displacement and ultimate bearing capacity of GFRP column. Second, in this paper, two optimal design schemes of GFRP columns are proposed. Third, for AEC industrial practitioners, the whole process is automatic, accurate and less reliant on data expertise and the optimization design scheme proposed in the article is relatively scientific.

## Introduction

Considering the physical limitations of concrete, composite structures of concrete have been introduced to extend the application area with the development of research materials. The application of glass fiber for reinforced concrete has further been promoted further significantly [1, 2].

GFRP, as a widely utilized material, has garnered the attention of numerous scholars in exploring various parameters that affect the properties of GFRP and concrete composite structure columns [3–10]. Fam and Rizkalla developed a parameter analytical model [3] to investigate the optimal structure of composite materials by examining the effects of different diameters of concrete-filled fiber reinforced columns on stress performance and failure mode. Shraideh and Aboutaha [4] discovered that reinforced concrete steel-GFRP sections possess high strength and good ductility by studying the amount, location, dimensions and mechanical properties of steel, GFRP and concrete in mixed concrete sections. A unique reinforcement technique for mixed concrete structures [5] was proposed by Mortazavi et al. and these scholars found that column pretension can be controlled by adjusting the amount of expansion material. Xue and Gong [6] suggested a new combination of columns to achieve better stress performance with a FRP columns which is composed of multiple materials. Ju et al. [7] studied the bending capacity of newly developed GFRP reinforced concrete composite columns through four-point bending experiments, discovering that the elastic modulus and mechanical properties of composite columns were improved compared with single GFRP columns. Zhang et al.’s research [8] focused on extrusion forming GFRP circular wrapped concrete column parameters’ effects in different sections on ultimate load bearing capacity, limit displacement, drawability, stiffness and the calculation formulas were proposed. Wang et al.’s [9] research explored how longitudinal reinforced beams affected structural failure mode and bending strength when combined with GFRP, and found that longitudinal reinforced beams performed better than transverse ones. Kusumawardaningsih and Hadi [10] conducted a comparative study on the performance of concentrated loads applied to fiber-reinforced polymer (FRP) and concrete columns with different cross sections, demonstrating that the round hole hollow columns perform better than the square hole ones. Xie and other scholars [11] performed experimental research on steel bone FRP tube concrete short columns under axial pressure, paid attention to the mechanical properties of short columns in relation to reinforcement ratio, concrete strength and GFRP column thickness. They proposed a design formula for concrete short columns under axial pressure considering their bearing capacity. Gemi et al. [12] investigated the stress performance of GFRP columns through experimental and analytical calculation, proposing a novel method for analyzing the beam bending performance, and providing a detailed damage analysis of the test specimens.

In these studies, the researchers derived laws and formulas based on numerous experiments, calculations and analyses. Before reaching the conclusion, multiple repeated experiments should be conducted to minimize errors. The significant consumption of human resources, materials and financial resources associated with each experiment necessitates careful consideration as flawed data will lead to formula deviation and questionable conclusions.

Model prediction with the machine learning method can reduce the number of experiments required while controlling costs and risks involved in such studies. Machine learning has been successfully employed across various fields to effectively address engineering problems [13–15]. In order to predict the run-in wear figure under the limited training sample size and analyze the impact of scene parameters on the run-in wear figure, Zhang et al. established a support vector machine regression model (SVMR) [13]. Kocak et al. proposed a multi-layer perceptron neural network model (MLPNN) [14] for predicting the compressive strength of cement slurry. The training and testing results of the model demonstrated its high accuracy and low error in predicting the compressive strength at 2, 7, 28, 56 and 90 days for cement mortars. Fatehi et al. proposed a design method [15] that combines machine learning model training, finite element data analysis, and a self-learning algorithm to investigate performance differences between high-performance ceramic structures and ordinary ceramic structures [16–23]. George and Cho established a multi-layer sensor regression model (MLPR) [16] to predict the physical characteristics of the shaking tank, and designed an optimal porous baffle to reduce liquid shaking in swinging and rectangular tank. Researchers have introduced Machine learning into civil structure optimization analysis. In order to predict the alkali tolerance of GFRP tendons and study the influence of environmental temperature and aging time on tensile strength retention of GFRP tendons, Mudassir Iqbal et al. established a random forest regression model [17]. Karimipour et al. using ANN method [18], conducted sensitivity analysis on GFRP performance by establishing a model that determined priority among input parameter and proposed a new high-precision formula. Yan et al. combined the non-linear mapping relationship of ANN and the global search ability of the genetic algorithm [19] to propose an optimization model to predict the bond strength of the GFRP columns. Aravind et al. [20] believed that machine learning methods provide more intuitive visualization for failure modes in composite columns, so they conducted a four-point bending test on the beams followed by image processing and fault mode recognition using six different machine learning methods. Bakouregui et al. [21] observed that XGBoost model performs better than other numerical models in predicting the bearing capacity of FRP-RC columns, so a high-precision XGBoost model was proposed to study the performance of columns. Dabiri et al. [22] employed machine learning methods and regression models to predict the displacement ductility ratio of reinforced concrete joints, resulting in a highly reliable prediction model. Basaran et al. [23] utilized various machine learning methods including ANN, MLP, and SVMR to investigate bond strength and development length of FRP columns.

However, existing studies on reinforced fiber materials and concrete mixed columns lack comparative analysis of different machine learning methods as well as optimization design based on optimal modeling method selection. In this paper, we propose utilized automatic machine learning methods for pattern recognition in the fiberglass polymer and concrete composite structure (GFRP) structures and subsequently achieving its optimized design.

## Methods and theory

### Tpot

Tpot, Tree-based Pipeline Optimization Tool, is a special algorithm in the field of machine learning. Tpot belongs to automatic machine learning and is a method based on tree and selection [24, 25]. Tpot algorithm essentially employs genetic programming technology to automate feature selection and model selection [26–28]. It can automatically perform feature selection, processing and construction while identifying the optimal algorithm combination from thousands of possibilities and optimize its parameters before outputting it as an optimal pipeline solution. Compared with ordinary machine learning algorithms, Tpot demonstrates significantly superior performance. [29, 30].

Tpot genetic algorithm can explore the selector, converter, classifier and return to implement any combination of machine learning pipeline. To enhance scalability of the algorithm and provide more interpretable results, a template can be specified for pipeline search at the beginning of each channel selector and a feature set selector can be added to divide the input dataset into smaller subsets. This enables genetic programming to select the best subset of the final pipe [31].

### MLP

Multi-layer Perceptron (MLP) is essentially a multi-layered neural network, which simulates human brain functions such as information reception, memory, and processing using interconnected neurons [32, 33]. MLP nervous system is a neural network with forward structure whose every node layer is fully connected to the next [32]. Nonlinear activation functions are applied to all neurons except for the input nodes. When these neurons are interconnected form a multi-layer perceptron, information is input into the input layer, transmitted to the hidden layer through function F processing, and then transmitted to the output layer through a multi-category logistic regression to complete information processing. Multi-layer perceptron algorithm is suitable for a great majority of scenes, such as speech processing, image processing and natural language processing, with high accuracy and it is easy to understand.

MLP neural network and composite models are normally used in industries to solve some complex problems like image recognition, radiation prediction and scene classification [34–36].

### SVM

Support Vector Machines (SVM) is a novel machine learning method in the field of modern data-driven modeling, characterized by its remarkable generalization performance as a linear classifier defined in feature space. It has been successfully applied in classification, regression and function estimation [37–39]. SVM possesses a solid mathematical theory and belongs to supervised learning models with related learning algorithms and it is a binary model. SVM is suitable for small sample learning and contains some nuclear skills, which help it to be a substantially nonlinear classifier [40]. The core idea of SVM model is to maximize the margin, transforming any problem into a convenient convex quadratic programming problem for optimal solution [41]. Therefore, SVM aims to provide an optimal answer for various problems.

SVM is often used in the construction industry to predict the performance of concrete [42, 43].

### Bagging

Bootstrap Aggregating (Bagging) algorithm was first proposed by Leo Breiman in 1996 and has been further refined since then [44]. Bagging serves as an ensemble learning method that can be combined with other machine learning algorithms to enhance accuracy and mitigate overfitting issues [45]. The main idea of bagging is to train several different models separately and aggregate their outputs through voting during testing. The specific operation is as follows. First of all, several groups of datasets are generated by self-help method (including put back sampling). Secondly, these groups of data sets are trained separately to obtain several classifiers. Finally, these classifiers are combined in using different strategies to obtain the final classifier. Bagging algorithm consistently improves the accuracy of learning algorithms within the field of machine learning.

### Random forest

The Random Forest algorithm is widely used in the field of machine learning employing multiple trees to process, train and predict data. The output category is determined by the majority category among individual trees comprising the Random Forest classifier. This algorithm was initially proposed by Leo Breiman and then calculated together with Adele Cutler [46]. The fundamental component of a Random Forest is a decision tree, which serves as a classifier. As there are as many classification results as there are decision trees in the forest, this algorithm exhibits high accuracy and robustness against outliers. Moreover, Random Forest is difficult to produce over fitting. Additionally, each decision tree only needs to consider a subset of candidate features in classification, s resulting in fast computation speed for both individual trees and the entire forest ensemble.

Random forests are frequently employed for optimizing and enhancing industrial concrete structures [47–49].

Table 1 presents the advantages, disadvantages, and characteristics of automatic machine learning as utilized in this article, as well as four other general machine learning approaches.

**Table 1:** Advantages and disadvantages of Tpot and four other machine learning methods.

	Tpot	MLP	SVM	Bagging	Random forest
*merit*	1.The Algorithm is simple and easy to understand. 2.High accuracy, and almost no need for manual parameter adjustment.	1.High identification rate. 2.Classification speed is fast. 3.Strong learning ability.	1.High generalization performance. 2.High-dimensional problems and non-linear problems can be solved. 3.The algorithm is simple and is insensitive to outliers.	1.The generalization error can be improved. 2.Sample selection has the same probability and high accuracy.	1.It can be trained in parallel, and the large sample data can train fast. 2.Strong generalization ability, easy to achieve.
*shortcoming*	1.The pipeline generation process is not clear, and it is not easy to explore deeply. 2.More parameters are required.	1.Easy to overfit. 2.The parameters are difficult to debug. 3.Study time is too long, and even may not achieve the learning purpose.	1.Large-scale samples could not be trained. 2.The kernel functions and parameters are difficult to select.	1. Performance is too dependent on the stability of the base classifier.	1.For some noisy datasets, it is easy to fall into overfitting. 2.Different values are easy to affect them.
*characteristic*	Using genetic algorithms, simple code can give the optimal machine learning model.	Each layer is fully connected and easy to fall into the local optimum.	It is a small-sample learning method that basically does not involve probability measures, etc., simplifying the general regression and classification problems.	Each sample was selected with the same probability and would not focus on any instance in the dataset.	The unbiased estimate of the internal error can be obtained in the generation process, and the model is easy to distinguish.

## Material and experiment

Experimental data on reinforced concrete winding GFRP columns were obtained from the study by scholars like Zhang et al. [8], while the experimental data on reinforced concrete filled GFRP columns were acquired from four articles by scholars including Kang He, Yu Chen, Wentao Xie Yuliang Guo and Xiaoyong Zhang [11, 50–52]. Permission was granted by these authors for utilizing their data.

**Fig 1:**
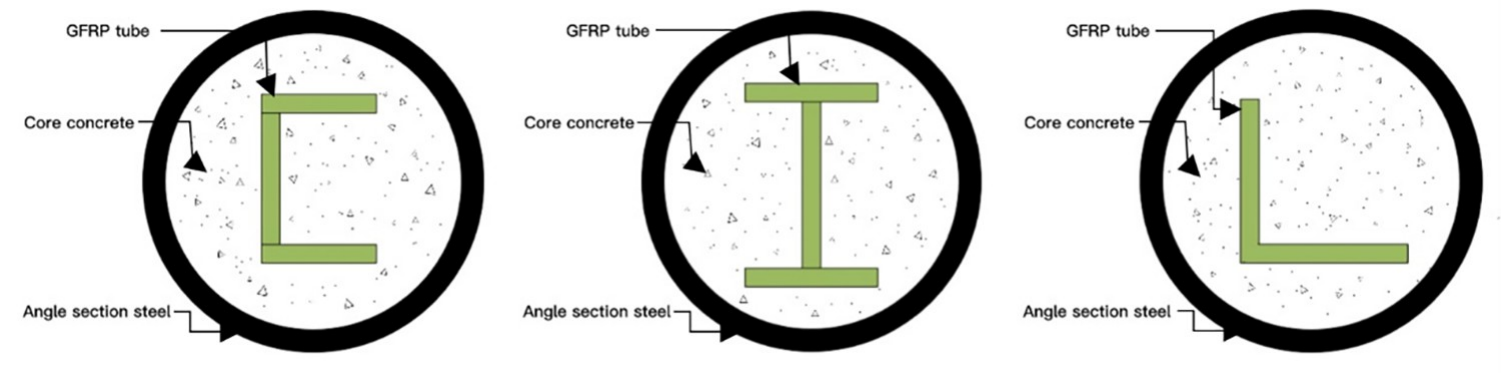
Sketch of the cross section of the reinforced concrete wound GFRP columns.

The influence of concrete winding GFRP columns on performance was investigated through axial compression tests and numerical simulations [8]. These columns consisted of pultruded glass fiber cloth (Angle section GFRP), channel steel pultruded glass fiber cloth (channel section GFRP) and I-shaped glass fiber cloth (I-section GFRP) (Fig 1). A total of 72 columns were tested, which were divided into 3 groups and each group of 24 columns that varied in terms of concrete strength, wound GFRP wall thickness and height. By employing the control variable method, each group was able to manipulate five variables, including cross-section shape of GFRP columns, concrete strength, thickness of GFRP column wall, inner diameter and column height. The experiment primarily focused on analyzing failure modes, ultimate bearing capacity, ductility and stiffness of columns under different variable values. Based on the mechanical model and test results of GFRP column constrained core concrete, a calculation formula for the ultimate bearing capacity of GFRP column was proposed.

**Fig 2:**
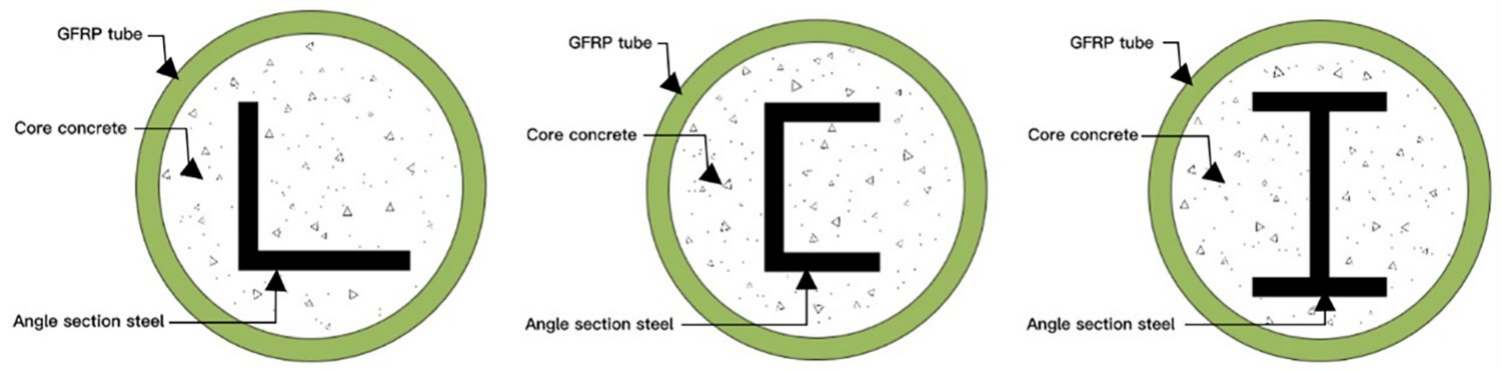
Sketch of the cross section of reinforced concrete filled GFRP columns.

The influence of different parameters on the performance of concrete filled GFRP columns under axial load was studied experimentally. Kang He et al. ‘s study [51] mainly targeted GFRP column with an L-shaped cross section type while Kang He and Yu Chen’s study [50] primarily aimed at GFRP column with a C-shaped cross section type. Wentao Xie and others mainly focused on GFRP columns with an I-shaped cross-sectional type [11]. Xiaoyong Zhang et al.’s investigation [52] examined GFRP columns filled with concrete without internal steel reinforcement material (Fig 2). The experiment without reinforcing material has 60 samples while each of the other three experiments mentioned above comprised 27 samples. These experiments thoroughly analyzed displacement-load curve, strain-load curve, ultimate bearing capacity, axial compression stiffness as well as failure modes exhibited by the samples. Consequently, optimal design formulas were proposed separately for three types of reinforced concrete-filled GFRP columns based on their respective cross-section shapes along with unreinforced GFRP columns.

**Fig 3:**
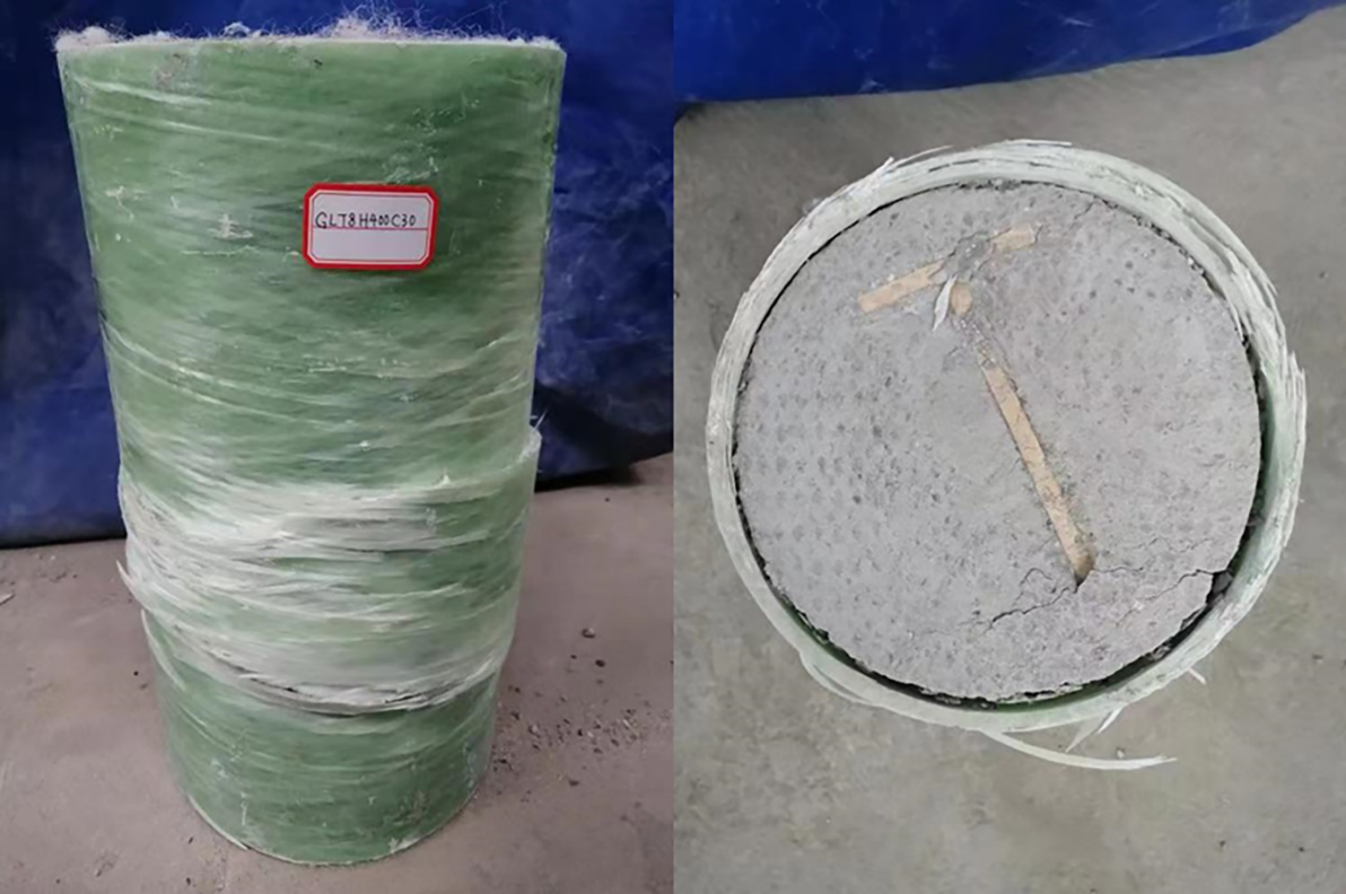
Physical diagram of the columns.

**Fig 4:**
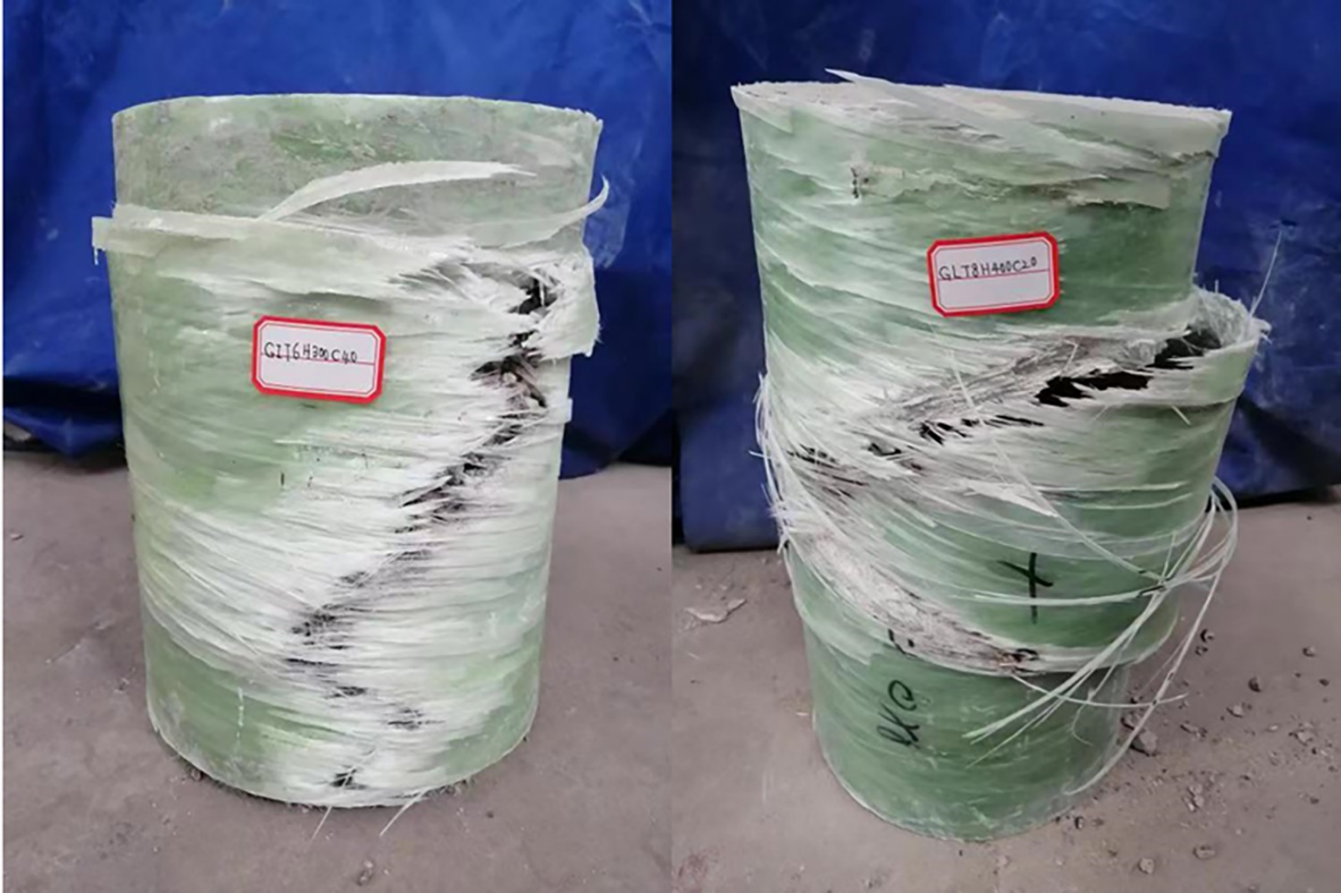
Column failure diagram.

Fig 3 depicts a section of the physical diagram illustrating the columns utilized in the aforementioned experiment, while Fig 4 illustrates its failure mode subsequent to the axial compression test.

## Methodologies

### Model establishment

In this study, the objective is to identify the most appropriate combination algorithm for machine learning and optimize its parameters using automatic machine learning Tpot genetic algorithm for a certain output parameter obtained from the experiment, in order to obtain the optimal Tpot model. In addition, the other four ordinary machine learning algorithms are used to establish the best model by tuning the parameters. The prediction models for the ultimate bearing capacity and ultimate displacement are established based on the aforementioned machine learning algorithms.

Description of input parameters during model construction: The symmetry ratio and area ratio are utilized to represent different cross-sectional patterns of concrete filled with GFRP columns in the experiment, with each cross-section’s symmetry ratio and area ratio being calculated as two separate input parameters. Additionally, the variable concrete strength (C) is also used as an input parameter. Furthermore, since the inner diameter of each experimental sample is the same, in order to reduce the input parameters to reduce the dimension of modeling and improve the accuracy, this paper incorporates the ratios of the height (H) to inner diameter (D) and wall thickness (T) to diameter (D), respectively, as additional input parameters. Hence, H/D and D/T ratios serve as two more input parameters. By selecting these five input parameters, all variables in the experiment can be adequately represented while minimizing modeling errors.

Description of output parameters during model construction: This paper, selects ultimate bearing capacity (Nr) and limit displacement (△u) as indicators for evaluating GFRP column performance. A higher ultimate bearing capacity indicates superior industrial performance of columns capable of supporting heavier loads. Conversely, a smaller limit displacement implies greater resistance against failure modes and longer service life for columns.

**Fig 5:**
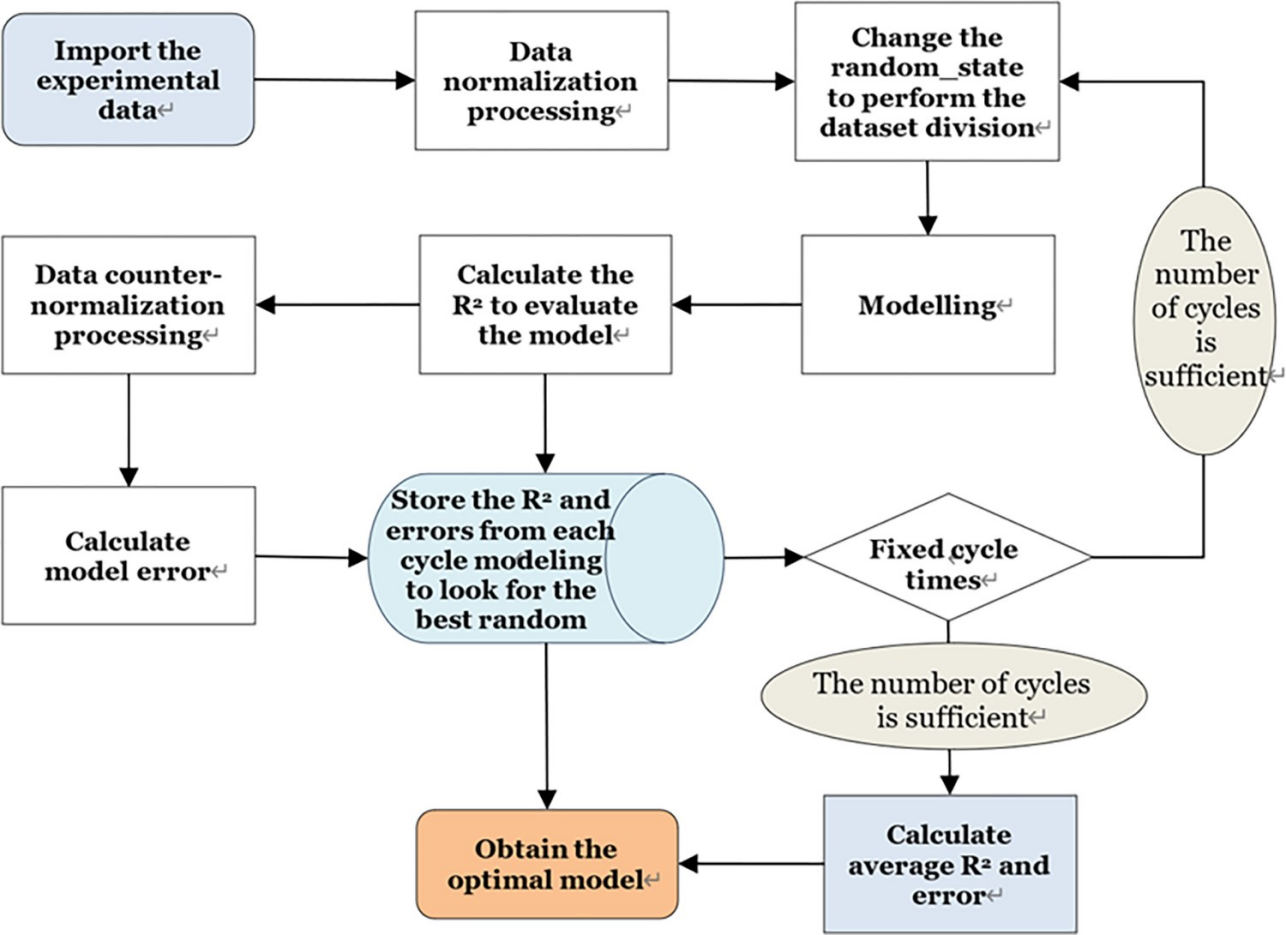
Modeling process.

When establishing the prediction model using experimental data, reinforced concrete wound GFRP column experiments were conducted at a 24:1 proportion where 69 out of the 72 groups were assigned as the training sets and the remaining 3 groups served as test sets. The experimental data for the reinforced concrete filled GFRP column was set with a ratio of 47:1 where 138 out of the total 141 groups of data are assigned as the training sets and the remaining 3 groups served as the test sets. Both Tpot automatic machine learning method and four other machine learning methods were employed to model the ultimate bearing capacity and ultimate displacement separately. The modeling process is illustrated in Fig 5. During this process, the training set and test set are arranged and combined by altering the random seed of the dataset. In this study, MAE (Mean Absolute Error) and R^2^ (Coefficient of Determination) are selected as evaluation metrics to identify the best model.

The platform utilized for modeling this article is Python, a high-level scripting language that combines interpretive, compilable, interactive, and object-oriented features.

The model building process is shown in the following figure (Fig 5):

The specific process is described as follows:

1. Import the data acquired from experiments.
2. Normalize the experimental data.
3. Divide the data.
4. Use Tpot, an automatic machine learning method, along with four other conventional machine learning methods to construct prediction models for two output characteristics: ultimate bearing capacity (Nr) and ultimate displacement (△u).
5. Evaluate the model quality by calculating R^2^ l after modeling.
6. Counter-normalized the predicted data to obtain corresponding real values of the output data.
7. Calculated MAE to facilitate a comprehensive evaluation of the model after obtaining the real data.
8. Average the experimental data for 100 times to prevent the influence of abnormal data on the results.
9. Vary random state of in partitioned datasets for arranging and combining training set and test set, repeating the above modeling process.
10. Store the R^2^ and MAE outputs from each cycle of modeling, identifying optimal random seed to determine best-performing model.
11. Conduct subsequent data testing on selected best model.

**Fig 6:**
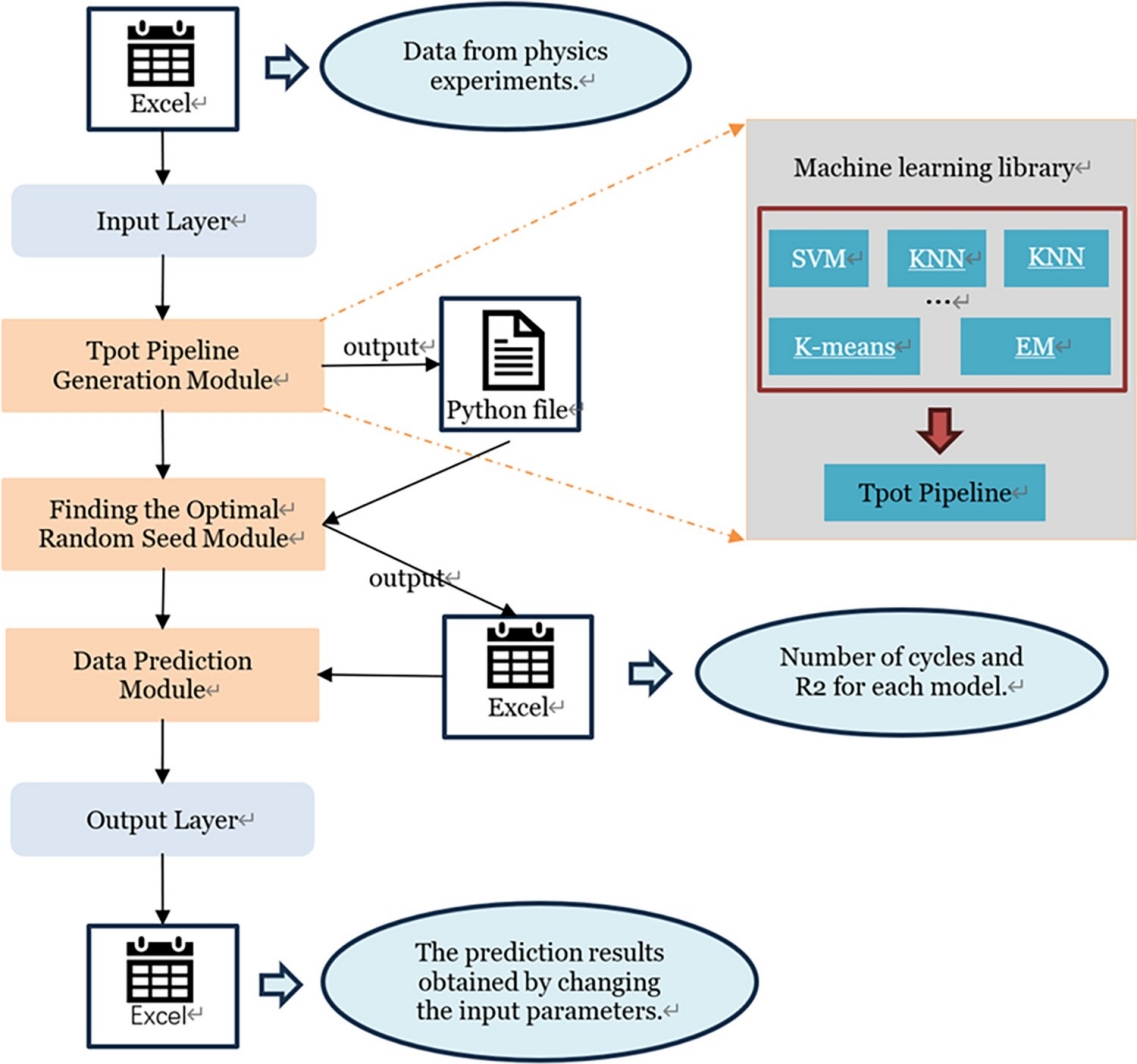
The network model architecture.

The network model architecture is shown in the following figure (Fig 6):

### Model validation and algorithm evaluation

In this paper, we select the absolute error (MAE) and the coefficient of determination (R^2^) to evaluate the model.

MAE means absolute error whose general expression is:

$$MAE=\frac{1}{n}\sum_{i=1}^{n}\left|\hat{y_{i}}-y_{i}\right|$$ (1)

A Mean MAE of 0 indicates perfect agreement between predicted and actual values, while a larger MAE signifies greater discrepancy between predicted and actual values, indicating poorer modeling performance. Conversely, a smaller MAE suggests better modeling effectiveness.

R^2^ measures how well the model fits the data. And its expression is:

$$R^{2}=1-\frac{\sum_{i}{\left(\hat{y_{i}}-y_{i}\right)}^{2}}{\sum_{i}{\left(\bar{y_{i}}-y_{i}\right)}^{2}}$$ (2)

The numerator component of the expression of R^2^ represents the error between the actual value and predicted value, reflecting the degree of fit of our model. The denominator component represents the error between the actual value and the average value, reflecting the deviation from mathematical expectation. By using R^2^, we can assess whether a model is good or bad. Its value ranges from 0 to 1, with higher values indicating better model performance.

### Model of reinforced concrete winding GFRP columns

Table 2 presents MAE and R^2^ resulting from modeling the ultimate bearing capacity Nr and ultimate displacement △u of reinforced concrete wound GFRP columns using different modeling methods, along with an evaluation of model quality. Different machine learning modeling methods are applicable for these two output parameters: SVM yields superior results based on MAE (0.36% for Nr) and R^2^ (0.9998 for Nr), as well as MAE (0.94% for △u) and R^2^ (0.9929 for △u). The average MAE across both parameters is 0.65%, while the average R^2^ is 0.9964%. Therefore, based on these data analyses, SVM demonstrates optimal performance in this study’s models. Next, the better result for the two parameters model is Tpot. For Nr, MAE is 1.53% and R^2^ is 0.9974. While for △u, MAE is 2.64% and R^2^ 0.9708. The two parameters have an average MAE of 2.085% and an average R^2^ of 0.9841. According to the data, the models work very well.

**Fig 7:**
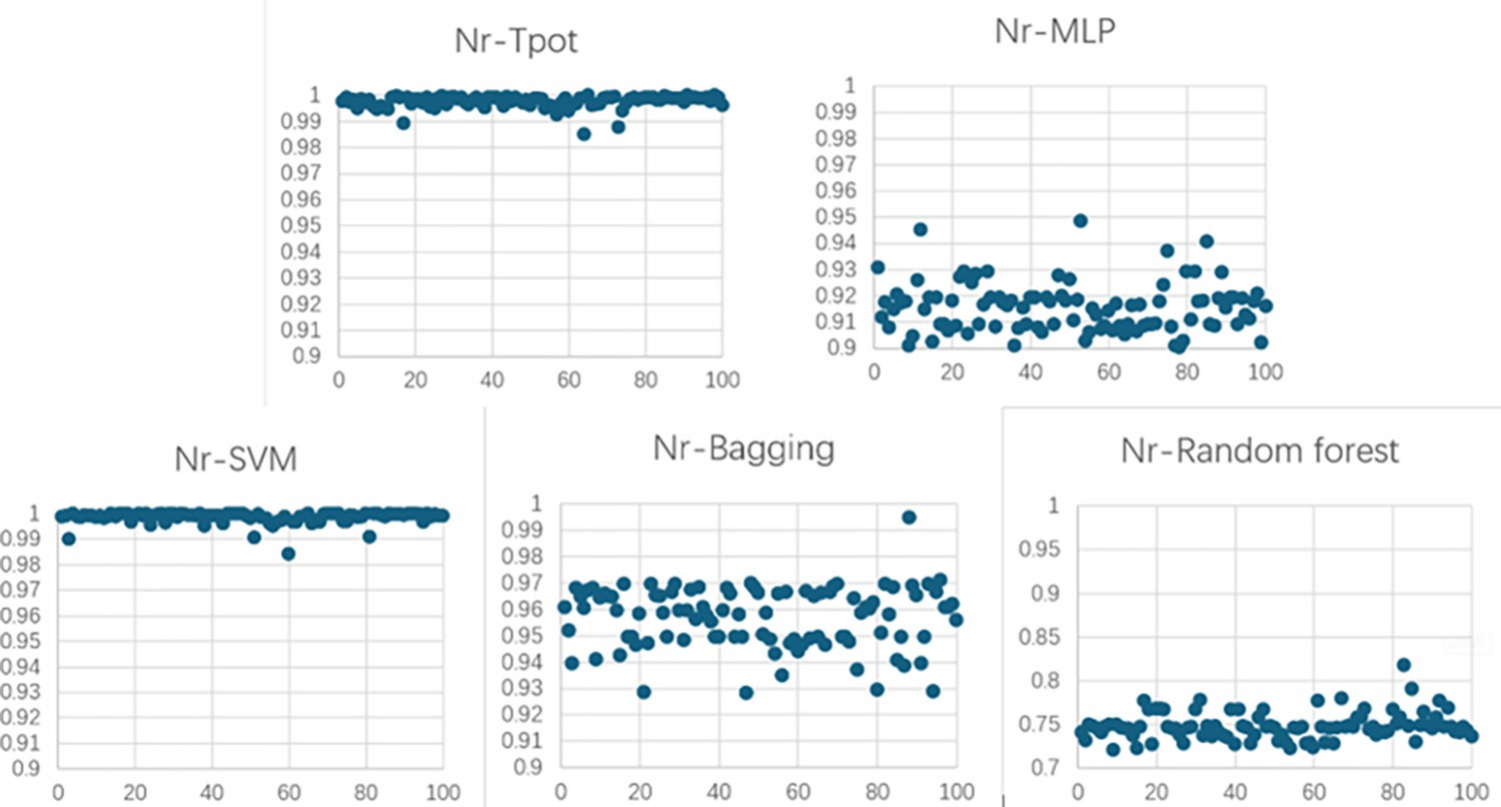
R^2^ of the models of concrete winding GFRP experiment for Nr.

**Fig 8:**
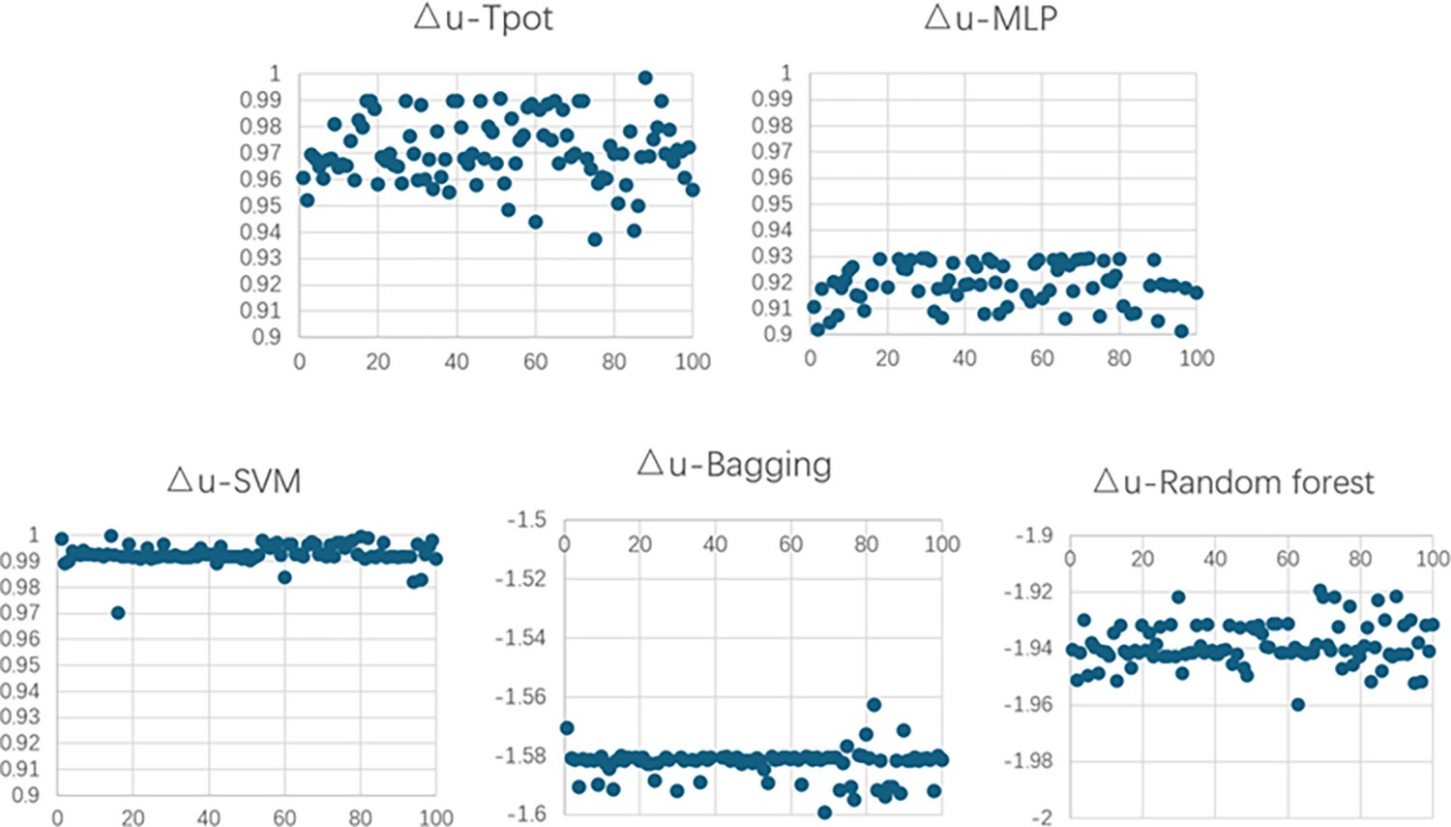
R^2^ of the models of concrete winding GFRP experiment for △u.

Figs 7 and 8 depict R^2^ of the models of concrete winding GFRP experiment.

**Table 2:** Evaluation of the models of concrete winding GFRP experiment.

MODELING EFFECT										
	Tpot		MLP		SVM		Bagging		Random forest	
	MAE	R^2	MAE	R^2	MAE	R^2	MAE	R^2	MAE	R^2
Nr	1.53%	0.9974	2.76%	0.9154	0.36%	0.9998	6.86%	0.9568	3.42%	0.7479
△u	2.64%	0.9708	2.55%	0.9144	0.94%	0.9929	7.26%	-1.5844	6.62%	-1.9389
Average value	2.085%	0.9841	2.655%	0.9149	0.65%	0.9964	7.06%	-0.3138	5.02%	-0.5955

### Model of GFRP columns filled with reinforced concrete

Table 3 presents the evaluation of different modeling methods for predicting ultimate bearing capacity (Nr) and ultimate displacement (△u) of GFRP column filled with reinforced concrete in terms of MAE and R^2^ values: SVM demonstrates the best performance for Nr with a minimal MAE of only 0.66% and an impressive R^2^ value of 0.9979; Random Forest ranks second with an MAE of 0.9174% and an R^2^ value of 0.9983; Tpot follows closely behind with an MAE of 1.378% and an R^2^ value of 0.9837 –all three models exhibit high accuracy levels. Among them, modeling with Tpot yields the best results for △u, with an MAE of only 0.1457% and an R^2^ value of 0.9987. SVM follows closely behind, achieving an MAE of 0.97% and an R^2^ value of 0.9955. Considering both parameters together, Tpot demonstrates superior modeling performance with an average MAE of 0.76185% and an average R^2^ value of 0.9962. Based on the average MAE and average R^2^ values for the two output parameters, SVM ranks second in terms of modeling effectiveness.

**Table 3:** Evaluation of the models of concrete filling GFRP experiment.

MODELING EFFECT										
	Tpot		MLP		SVM		Bagging		Random forest	
	MAE	R^2	MAE	R^2	MAE	R^2	MAE	R^2	MAE	R^2
Nr	1.378%	0.9837	1.985%	0.9883	0.66%	0.9979	2.786%	0.9582	0.9174%	0.9983
△u	0.1457%	0.9987	1.983%	0.9212	0.97%	0.9955	4.36%	0.854	2.173%	0.9832
Average value	0.76185%	0.9962	1.984%	0.9548	0.815%	0.9967	3.573%	0.9061	1.5452%	0.9908

**Fig 9:**
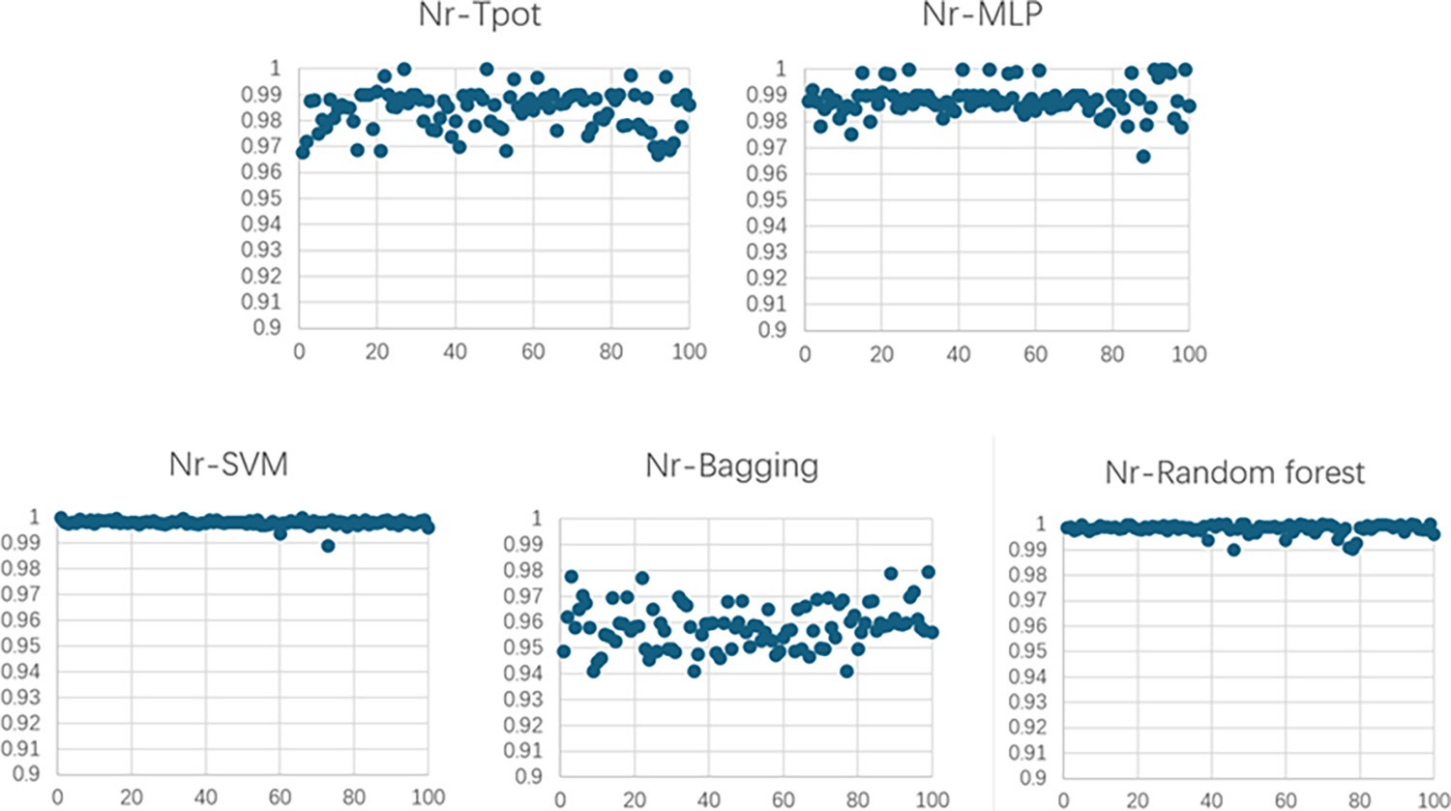
R^2^ of the models of concrete filling GFRP experiment for Nr.

**Fig 10:**
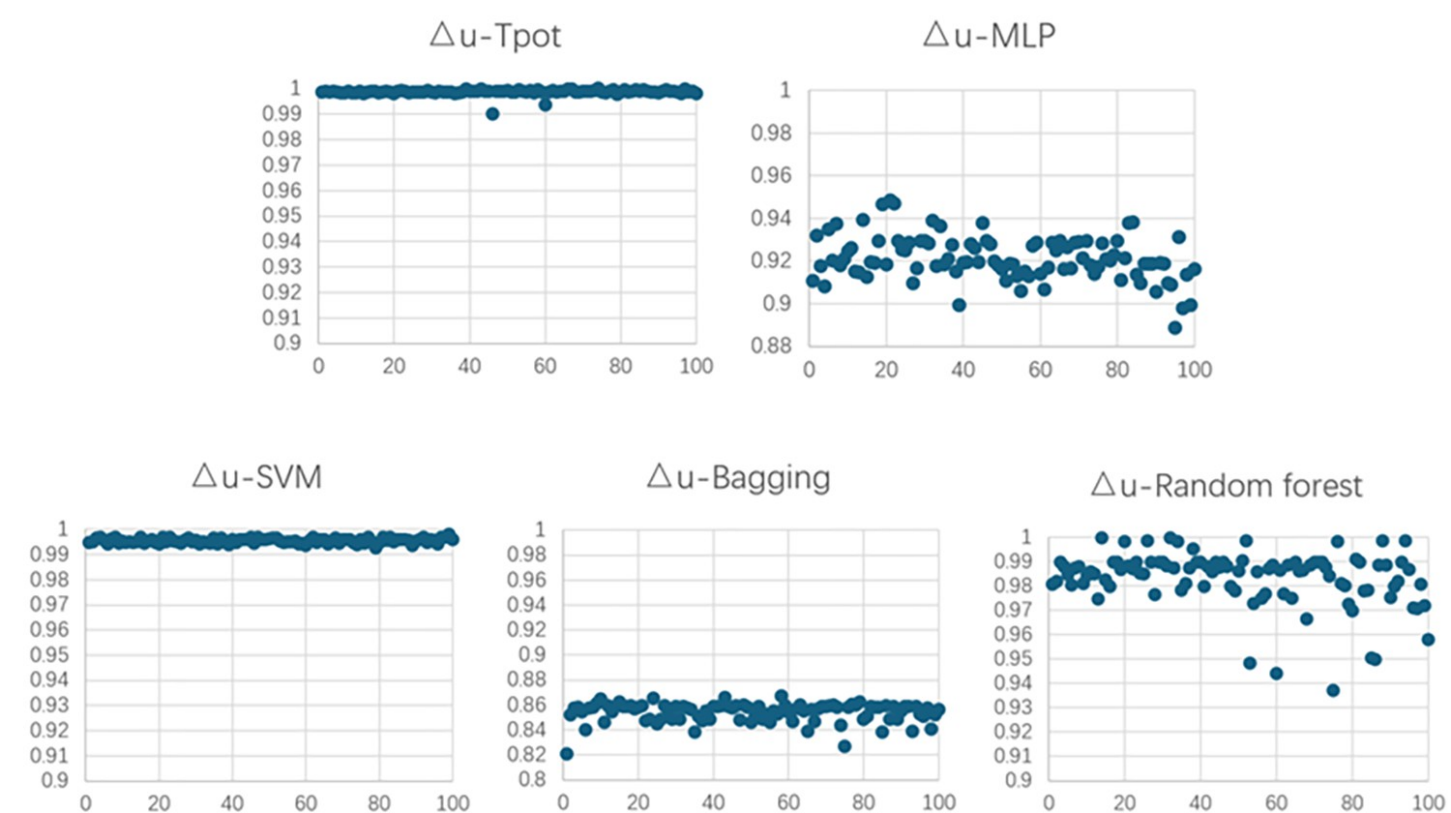
R^2^ of the models of concrete filling GFRP experiment for △u.

Figs 9 and 10 depict R^2^ of the models of concrete filling GFRP experiment.

## Results and discussion

In order to investigate the influence of individual parameter, we systematically increase each input parameters while keeping other values constant at equal intervals within their respective ranges. Taking H/D as an example, we incrementally vary its test input values to obtain eleven data groups based on ten equal divisions between the maximum and minimum values while fixing other parameters at predetermined values. Subsequently, these new data were input into the model and run repeatedly for 100 times to output the predicted Nr and △u from which we calculate their averages to minimize MAE. We repeat this process by sequentially increasing each remaining parameter using equal divisions within their respective ranges to conduct five tests in order to comprehend how all input parameters affect ultimate bearing capacity and ultimate displacement of columns.

### Experiment of reinforced concrete wound GFRP columns

By considering both MAE and R^2^ metrics together, it becomes evident that SVM model has the best effect, followed by Tpot.

SVM is a representative small sample learning method known for its significant generalization performance, especially when dealing with limited data samples. Tpot stands out as well due to its automatic machine learning approach that explores optimal subsets among various optimization pipelines. Its high accuracy makes it suitable for all small sample datasets comprehensively. However, Bagging and Random Forest, which are also machine learning methods suitable for small sample research, are not appropriate to be applied to this paper. Bagging heavily relies on the stability of classifier, while Random forest is more suitable for large sample data research.

**Fig 11:**
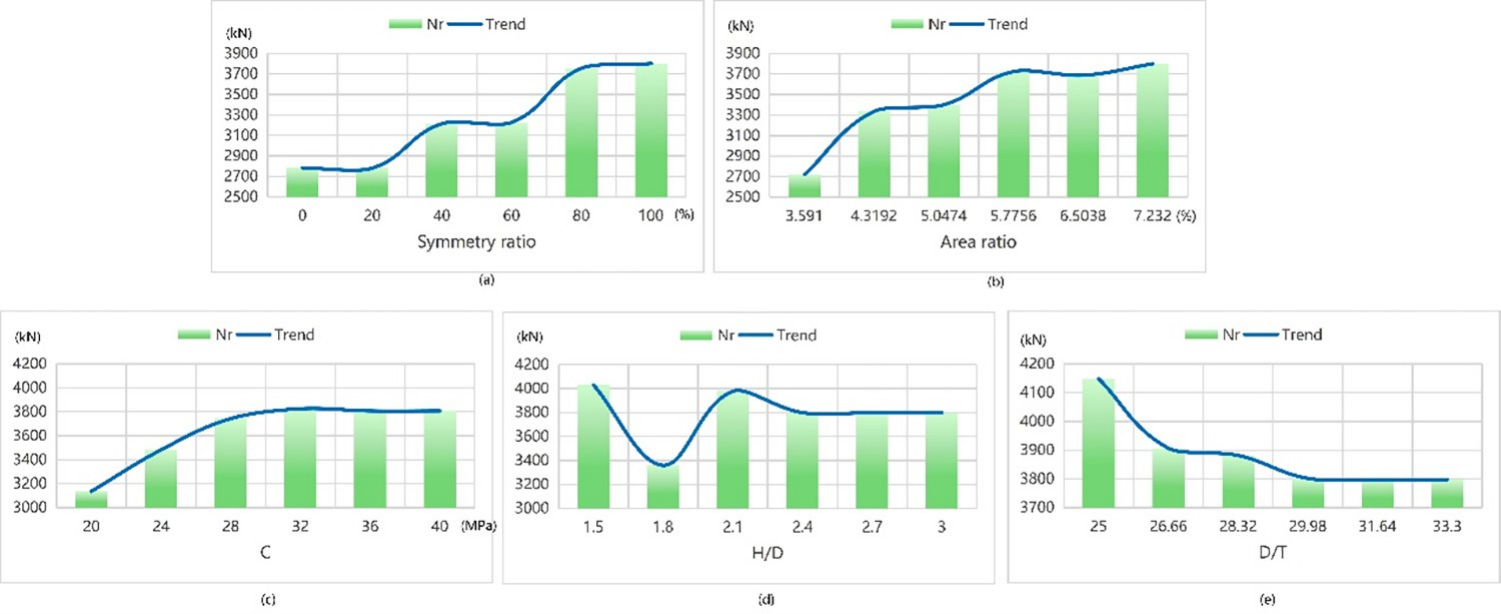
The law of ultimate bearing capacity affected by each parameter.

**Fig 12:**
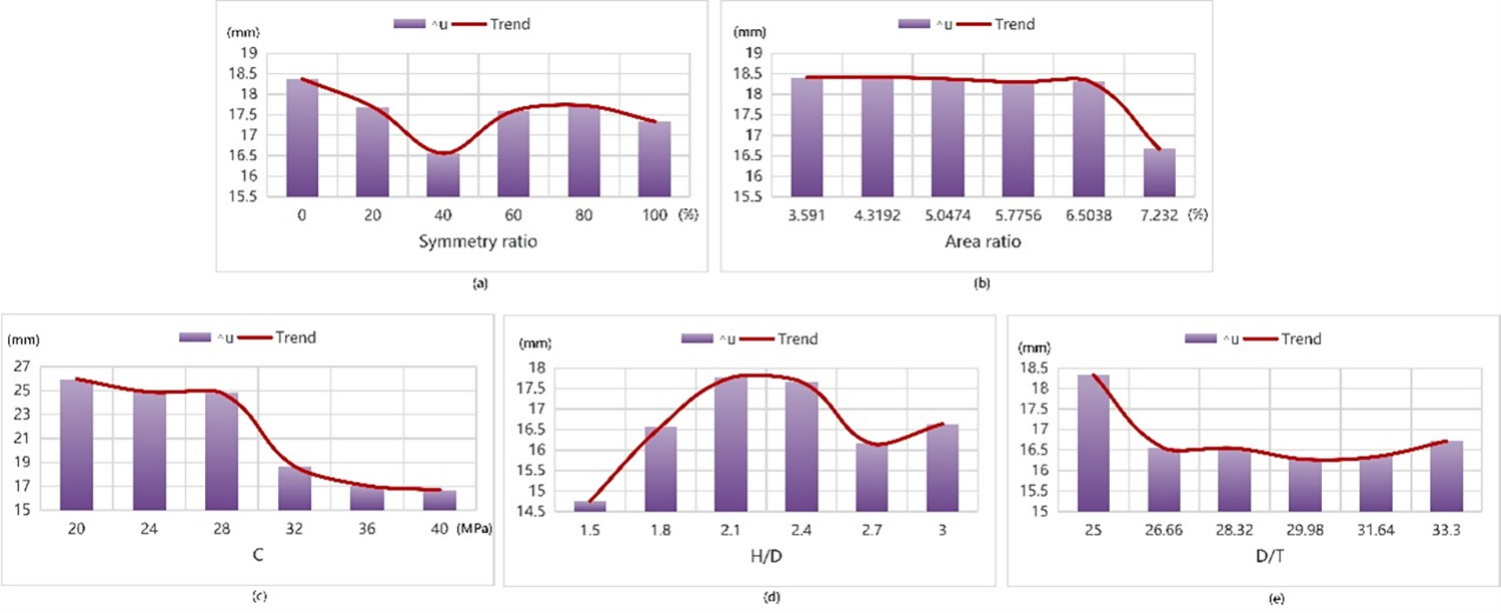
The law of ultimate displacement influenced by each parameter.

The data test results obtained using Tpot optimal model are shown presented Figs 11 and 12. Although SVM has a smaller overall error and R^2^ closer to 1, it is considered too simplistic as an algorithm. During the process of data calculation, it was observed that the predicted value by SVM has a large deviation from the real value, which is inconsistent with the real situation. In the study of Aravind N et al. [20], six machine learning classifiers were used to identify fault modes, and the results showed that SVM had the best effect with 100% accuracy. While SVM is a small sample learning method, which can solve high-dimensional and nonlinear problems, but it is extremely insensitive to data outliers. In addition, the efficiency of SVM is too low in solving the problems of multiple classification, which is not applicable to this paper. The reason for this is that the kernel function chosen by the SVM model possesses excessive power, leading to overfitting of the model. Consequently, we have adopted the Tpot optimal model as our preferred choice due to its supported prediction results based on experimental data.

When gradually increasing within its interval range of symmetry ratio, Nr exhibits an upward trend while △u presents a slow downward trend with a change pattern of sharp decline followed by increase in its middle interval range. When the symmetry ratio reaches 40%, we observe that ultimate displacement attains its minimum value at 16.56 mm–representing an optimal state according to our model’s analysis. In contrast, when reaching a symmetry ratio of 100%, maximum ultimate bearing capacity can be achieved at 3800 KN.

When the area ratio increases gradually, the curve of Nr exhibits an overall increasing trend with a step-like shape in the middle section. The curve of △u initially levels off and then falls. At an area ratio of 7.232%, the ultimate displacement is minimized to only 16.67 mm, indicating the optimal state of the model. Additionally, at this time, the ultimate bearing capacity reaches its maximum of 3799 kN.

When the concrete strength increases gradually within its range, the Nr curve initially presents an ascending trend followed by a gradual stabilization. The △u curve stabilizes and then experiences a temporary drop before leveling off again. At a concrete strength of 40 MPa, the ultimate displacement reaches its minimum value of 16.69 mm, indicating the optimal state of the model. Simultaneously, the ultimate load also attains its maximum value of 3807 kN. As H/D progressively increases, the first half of the Nr curve oscillates while the second half tends to stabilize. The △u curve rises gradually at first, then decreases slowly after reaching the peak value, and then slowly rises again. At an H/D ratio of 1.5, the ultimate displacement reaches its minimum at 16.64 mm, signifying an optimal state for the model with a maximum load-bearing capacity of 4000 kN achieved during this condition.

When D/T gradually increases within its range, the curve of Nr presents a declining state as a whole with a step shape in the first half and a relatively stable trend in the end. The curve of △u presents a declining trend as a whole which rises and then declines with a small amplitude in the middle and gradually rises ultimately. At D/T values of approximately 29.98 and 25 respectively; we observe that these conditions correspond to minimum limit displacements (16.27 mm) and maximum ultimate bearing capacities (4147 kN).

The above results indicate that the following:

1. The greater the symmetry ratio is, the greater Nr is. However, there is little change in Nr between 80% and 100%. When the symmetry ratio is around 40%, △u the smallest.
2. The larger the area ratio is, the larger Nr is and the smaller △u is
3. The greater the concrete strength is, the greater Nr is and the smaller △u is. But after the concrete strength rises to 30 MPa, Nr almost has no change.
4. The smaller H/D is, the smaller △u is. And Nr reaches the maximum at 1.5 and 2.1.
5. The smaller D/T is, the greater Nr is. And △u is the smallest between 26.66 and 31.64.

When aiming for maximum limit load capacity, is recommended to have an asymmetrical ratio of 100%, an area ratio of 7.232%, a concrete strength of 30 MPa, an H/D of 1.5, and a D/T of 25. Considering that the area ratio and symmetry ratio have the greatest influence on the ultimate load, when the area ratio of 7.232% conflicts with the requirement of H/D of 1.5, the area ratio condition is given priority. Therefore, the test results of the model are consistent with the maximum ultimate load of 4833.2 kN of the experimental specimen numbered GIT8H600C30 [8]. The test number GIT8H600C30 device has a concrete strength of 30 MPa, an H/D of 3, a D/T of 25, and the cross-section is type I, that is, the symmetry ratio is 100%, and the area ratio is 7.232%.

On the other hand, to meet the minimum limit displacement requirement, it is suggested to have an asymmetric ratio of 40%, an area ratio of 7.232%, a concrete strength of 40 MPa, an H/D of 1.5, and a D/T of 33.3. Considering that the concrete strength and H/D have the greatest influence on the ultimate load and the symmetric ratio of 40% and the area ratio of 7.232% cannot be met at the same time, columns shaped as C and I in cross-section are selected for analysis. The results are consistent with the experiments numbered GCT6H300C40 and GIT6H300C40 respectively [8], whose ultimate displacements are 15.27 mm and 14.87 mm. The experiment number GCT6H300C40 device has a concrete strength of 40MPa, an H/D of 1.5, a D/T of 33.3, and a cross-section of C, that is, a symmetry ratio of 53.191% and an area ratio of 3.591%. The test number GIT6H300C40 device has a concrete strength of 40 MPa, an H/D of 1.5, a D/T of 33.3, and a cross section of type I, that is, a symmetry ratio of 100%, and an area ratio of 7.232%.

Considering the ultimate bearing capacity, the optimal performance is achieved when the cross-sectional figure of concrete filled with wound GFRP column is symmetrical. This is because under the condition of uniform stress, when the cross section of the GFRP material is symmetrical, there will be no collapse due to excessive stress and failure of the weak point of the support. Additionally, it is not necessary for the area ratio to be excessively large under these circumstances considering cost factors; hence other shapes with larger area ratios than those observed in this experiment will not be considered temporarily.

### Experiment of GFRP columns filled with reinforced concrete

Combining MAE and R^2^, it can be concluded that Tpot automatic machine learning method yields superior modeling results for the two parameters, followed by SVM. Therefore, Tpot model is selected for data prediction ultimately.

**Fig 13:**
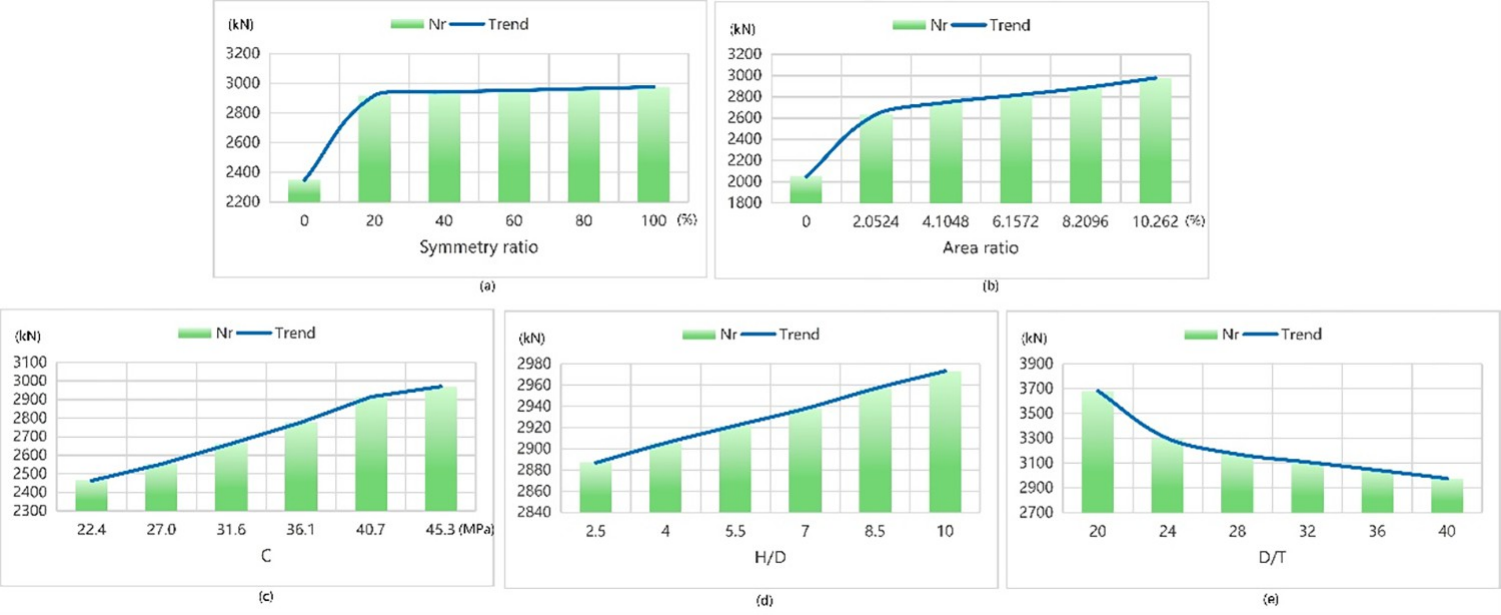
The law of ultimate bearing capacity affected by each parameter.

**Fig 14:**
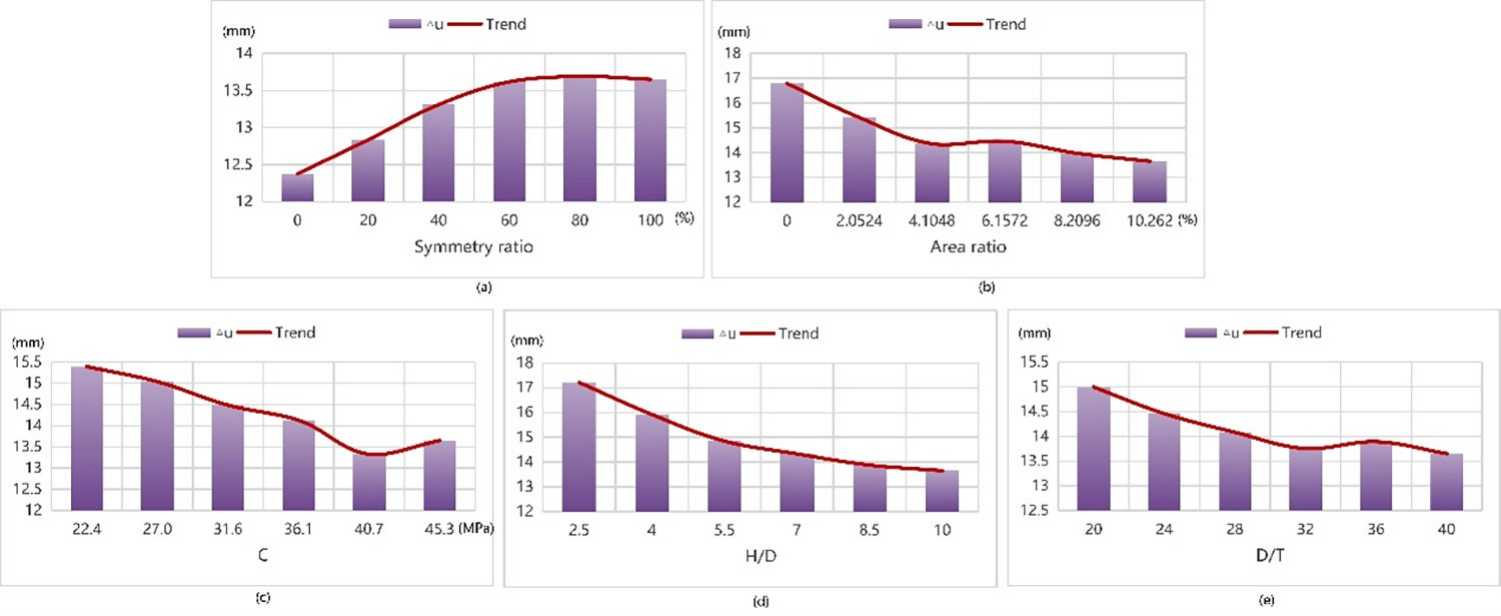
The law of ultimate displacement influenced by each parameter.

The following (Figs 13 and 14) are the results obtained from data testing with the Tpot best model.

As the symmetry ratio gradually increases in its range interval, the curve of Nr initially rises before stabilizing, while the curve of △u experiences gradual initial increase followed by minimal changes after reaching the peak value. When the symmetry ratio reaches approximately 20%, the ultimate load attains its peak value around 2950 kN without significant fluctuations thereafter. Conversely, when the symmetry ratio is 0, the value of the ultimate displacement remains minimal at only 12.38 mm. And the model presents the best state at the time.

When the area ratio increases gradually within its range interval, the Nr curve exhibits a rising trend on the whole whose rising range is large at the beginning, and then gradually decreases. The △u curve presents a declining trend on the whole, with a table-like shape in the middle and there is a period of slow recovery whose amplitude is small and then decreased again. The model reaches its optimal state when the area ratio is at its maximum of 10.262%, resulting in a peak ultimate load of 3000 kN and minimum ultimate displacement of 13.65 mm.

As the concrete strength gradually increases within its range, the curve of Nr generally shows an upward trend with a larger increase initially followed by a slightly smaller increase later on the first half of △u curve decreases, eventually showing some slow recovery but with limited amplitude. When the concrete strength reaches 40.72 MPa, the limit displacement is minimized at 13.32 mm, and when the concrete strength is the maximum of 45.3 MPa, the ultimate load reaches the maximum of 2970 kN, and the model presents the best state. When H/D gradually increases in its range, the Nr curve generally presents a rising trend, and the rising range has little change. While the △u curve generally presents a declining trend with a large decline in the first half and a slow decline in the second half. When H/D is 10, the ultimate load reaches the maximum of 2973 kN and the limit displacement is the minimum of 13.65 mm. The model presents the best state.

When H/D gradually increases within its range, the curve of Nr presents an ascending trend without significant changes in rising range. While the curve of △u is generally declining and presents a platform change in the second half with a slight rise and then decline. When H/D equals to 20, the ultimate load reaches the maximum of 3700 kN, while when H/D is 40, the ultimate displacement is the minimum of 13.65 mm, and the model reaches the best state.

The above results indicate that the following:

1. The larger the symmetry ratio is, the greater Nr and △u of the column are. However, Nr is almost unchanged in the range of 20% to 100%, and △u is almost unchanged in the range of 60% to 100%.
2. The larger the area ratio is, the greater Nr is and the smaller △u is.
3. The greater the concrete strength is, the larger Nr is and the smaller △u is.
4. The larger the H/D is, the larger Nr is and the smaller △u is.
5. The larger the D/T is, the smaller Nr and △u are.

To achieve maximum ultimate load bearing capacity, we recommend using a symmetric ratio between 20% to 100%, an area ratio of 10.262%, a concrete strength of 45.3 MPa, an H/D of 10 and a D/T of 20. When the area ratio is in conflict with H/D, the area ratio condition is given priority. The results of model experiment are consistent with the results of test piece *α*20-*β*0.070-C40 [50] with the maximum ultimate load reaching 3914.9 kN. The experimental device has a concrete strength of 45.3 MPa, an H/D of 2.5, a D/T of 20, and a cross section of C type, that is, the symmetry ratio is 22%, and the area ratio is 8.611%.

For minimum limit displacement requirements, a symmetric ratio of 0, is recommended along with an area ratio of 10.262%, a concrete strength of 40.72MPa, an H/D of 10 and a D/T of 40. There are no specimens with both area ratio and symmetry ratio satisfied, so type C and type without internal reinforcement are selected for analysis. The results are consistent with the results of 11.5 mm in experiment number 40–0.022-C40 and 8.73 mm in experiment number GT5-H2.00-L400 [52]. The experiment number 40–0.022-C40 device has a concrete strength of 42.5 MPa, an H/D of 2.5, a D/T of 40, and a cross-section of L type whose symmetry ratio is 0% and area ratio is 2.424%. The test number GT5-H2.00-L400 device has a concrete strength of 30.7MPa, an H/D of 10, a D/T of 40, and the cross-section is L-shaped, that is, the symmetry ratio is 0%, and the area ratio is 0%.

### Design and optimization schemes

It is necessary to acknowledge the limitations of this study, as our model has been trained on a limited number of samples and is restricted to the L-type, I-type and C-type columns mentioned in this paper. Therefore, the optimization design presented in this paper is selected from these three types of GFRP tubular columns.

Optimization results and suggestions of concrete wound GFRP column: Considering the range of training data available, our model suggests that the optimal design choice is an I-type column with a concrete strength is set at 40 Mpa, H/D set at 1.5, and D/T set at 25. The failure index is expected to be D/T. According to the law in the above figure, the ultimate bearing capacity and the ultimate displacement conflict in the D/T setting size, and the ultimate bearing capacity is given priority to the performance of the column.

Optimization design scheme of concrete-filled GFRP column: Taking into account the range of training data used in our model, we propose that a C-type column would be most suitable with a concrete strength set at 45.3 MPa, H/D set at 10, D/T set at 20. The failure index is expected to be D/T.

The predicted data generated by our model a align closely with the experimental data, and the model has high precision which makes it suitable for industrial optimization designs.

## Conclusions

This article is based on limited samples with intricate structure and employs automatic machine learning methods to investigate circular reinforced concrete wound GFRP columns and reinforced concrete filled GFRP columns. The primary focus lies in examining the influence of concrete strength, thickness of wound GFRP columns, inner diameter and height of columns posts and different cross-sectional shapes of internal material of columns on the performance of columns. The failure mode and ultimate load carrying capacity of the columns are analyzed and prediction models are established to predict and analyze the optimal parameter value. The predicted results are effectively supported by the experimental data. Finally, the optimal design and optimization scheme of two kinds of GFRP columns are given in this article.

The following conclusions can be drawn from this paper:

1. As a high-performance and easy-to-use automatic machine learning model, Tpot is proved to be efficient and accurate in this paper, and its performance is better than other four machine learning methods.
2. The effects of symmetry ratio, area ratio, concrete strength C, H/D and D/T on the properties of the column are determined, and the optimal values for each parameter are obtained.
3. The paper puts forward the specific optimization design scheme:
  - For concrete wound GFRP columns—I-type column with a concrete strength set at 40 Mpa, H/D set at 1.5, and D/T set at 25.
  - For concrete-filled GFRP columns—C-type column with a concrete strength set at 45.3 MPa, H/D set at 10, D/T set at 20.

The contribution of this study to knowledge is mainly in structural engineering, but also includes a small number of calculations. Our work is based on small sample learning, so our final optimal design is based on the three cross-sectional columns described in the paper. Therefore, whether the conclusions from the Tpot model can be applied to the optimal design of random cross-sectional GFRP column is worthy of further discussion.
